# Survival and quality‐of‐life implications of cytopenia trajectories in ruxolitinib‐treated myelofibrosis

**DOI:** 10.1002/cncr.70320

**Published:** 2026-02-13

**Authors:** Francesca Palandri, Giovanni Caocci, Elisabetta Abruzzese, Mario Tiribelli, Erika Morsia, Mirko Farina, Giulia Benevolo, Eloise Beggiato, Bruno Martino, Novella Pugliese, Alessia Tieghi, Monica Crugnola, Gianni Binotto, Francesco Cavazzini, Alessandra Iurlo, Alessandro Isidori, Alessandra Dedola, Emilia Scalzulli, Andrea Duminuco, Daniele Cattaneo, Roberto M. Lemoli, Costanza Bosi, Daniela Cilloni, Monica Bocchia, Fabrizio Pane, Chiara Sartor, Florian H. Heidel, Massimo Breccia, Filippo Branzanti, Giuseppe A. Palumbo, Massimiliano Bonifacio, Elena M. Elli

**Affiliations:** ^1^ IRCCS Azienda Ospedaliero‐Universitaria di Bologna Istituto di Ematologia “Seràgnoli” Bologna Italy; ^2^ Ematologia, Ospedale Businco Università degli studi di Cagliari Cagliari Italy; ^3^ Division of Hematology Ospedale S. Eugenio Rome Italy; ^4^ Division of Hematology and Bone Marrow Transplantation, Department of Medical Area University of Udine Udine Italy; ^5^ Hematology Unit Department of Clinical and Molecular Sciences DISCLIMO Università Politecnica delle Marche Ancona Italy; ^6^ Unit of Blood Diseases and Stem Cells Transplantation, Department of Clinical and Experimental Sciences University of Brescia ASST Spedali Civili of Brescia Brescia Italy; ^7^ University Hematology Division Città della Salute e della Scienza Hospital Turin Italy; ^8^ Unit of Hematology Department of Oncology University of Torino Turin Italy; ^9^ Division of Hematology Azienda Ospedaliera “Bianchi Melacrino Morelli” Reggio Calabria Italy; ^10^ Department of Clinical Medicine and Surgery Federico II University Medical School Naples Italy; ^11^ Department of Hematology Azienda USL‐IRCCS di Reggio Emilia Reggio Emilia Italy; ^12^ Haematology and Bone Marrow Transplantation Centre Azienda Ospedaliero‐Universitaria di Parma Parma Italy; ^13^ Unit of Hematology and Clinical Immunology University of Padova Padua Italy; ^14^ Division of Hematology University of Ferrara Ferrara Italy; ^15^ Hematology Division Foundation IRCCS Ca' Granda Ospedale Maggiore Policlinico Milan Italy; ^16^ Hematology and Stem Cell Transplant Center AORMN Hospital Pesaro Italy; ^17^ Dipartimento di Scienze Mediche e Chirurgiche Università di Bologna Bologna Italy; ^18^ Hematology Department of Translational and Precision Medicine Az. Policlinico Umberto I‐Sapienza University Rome Italy; ^19^ UO Ematologia AOU Policlinico “G. Rodolico”‐San Marco Catania Italy; ^20^ Clinic of Hematology, Department of Internal Medicine University of Genoa Genoa Italy; ^21^ IRCCS Policlinico San Martino Genoa Italy; ^22^ Division of Hematology AUSL di Piacenza Piacenza Italy; ^23^ Department of Clinical and Biological Sciences University of Turin Turin Italy; ^24^ Hematology Unit Azienda Ospedaliera Universitaria Senese University of Siena Siena Italy; ^25^ Hematology, Hemostasis, Oncology and Stem Cell Transplantation Hannover Medical School Hanover Germany; ^26^ Department of Scienze Mediche Chirurgiche e Tecnologie Avanzate “G.F. Ingrassia” University of Catania Catania Italy; ^27^ Department of Engineering for Innovation Medicine, Section of Innovation Biomedicine Hematology Area University of Verona Verona Italy; ^28^ Fondazione IRCCS San Gerardo dei Tintori Divisione di Ematologia e Unità Trapianto di Midollo Monza Italy

**Keywords:** anemia, cytopenia, myelofibrosis, ruxolitinib, survival, thrombocytopenia

## Abstract

**Background:**

Cytopenia is a common complication in patients with myelofibrosis and may worsen during treatment with ruxolitinib.

**Methods:**

The RUX‐MF multicenter study evaluated 879 patients treated with ruxolitinib for at least 6 months, categorizing them into four groups based on the evolution of cytopenia: never cytopenic, treatment‐emergent cytopenia, persistent cytopenia, and improved anemia.

**Results:**

At baseline, 40.6% of patients presented with cytopenia, increasing to 57.8% after 6 months. Baseline cytopenia was associated with significantly reduced median overall survival (OS) compared to noncytopenic patients (3.7 vs. 6.7 years). Prognosis varied notably across groups: patients who remained noncytopenic had the median best OS (8.1 years), whereas those with persistent cytopenia had the worst (3.7 years). Treatment‐emergent cytopenia was linked to intermediate outcomes (5.1 years), with isolated thrombocytopenia showing the poorest prognosis (4.3 years) and anemia a slightly better one (6.1 years). Patients with improved anemia had better survival than those with persistent anemia (5.2 vs. 3.5 years). Symptom response mirrored survival trends, with the best outcomes in noncytopenic and improved anemia groups.

**Conclusions:**

These findings highlight the prognostic significance of cytopenia dynamics during ruxolitinib therapy and support the use of cytopenia trajectory monitoring as a valuable tool for risk stratification and treatment optimization in myelofibrosis.

## Abstract 2

Cytopenia trajectories during ruxolitinib therapy in myelofibrosis provide strong prognostic information, with persistent or treatment‐emergent cytopenias associated with inferior survival. Hemoglobin recovery identifies a small subgroup with improved survival and quality of life.

## INTRODUCTION

Myelofibrosis (MF) is a chronic myeloproliferative neoplasm characterized by bone marrow fibrosis, extramedullary hematopoiesis, and a variable clinical course.^1^ Cytopenia, mainly anemia and thrombocytopenia, are common manifestations of MF, resulting from both disease‐related bone marrow failure and therapeutic interventions.^2^, ^3^, ^4^

Cytopenia at diagnosis remains a recognized adverse prognostic marker in myelofibrosis, associated with inferior overall survival and elevated risk of leukemic transformation.^5^, ^6^, ^7^, ^8^, ^9^

The management of MF has evolved significantly with the introduction of Janus kinase (JAK) inhibitors, particularly ruxolitinib, which remains the standard of care for symptomatic, higher‐risk MF. Ruxolitinib has demonstrated efficacy in reducing splenomegaly and improving quality of life, but it does not ameliorate cytopenia and, in some cases, may exacerbate them.^10^, ^11^

Previous findings further supported that anemia at ruxolitinib initiation and/or discontinuation may confer increased vulnerability to disease progression.^12^, ^13^, ^14^, ^15^

The adverse prognostic significance of baseline and of early‐onset or worsening anemia under ruxolitinib treatment has also been captured and expanded in dynamic prognostic frameworks such as RR6 and iRR6, which incorporate transfusion needs and early hematologic dynamics as pivotal predictors of clinical outcomes.^16^, ^17^

A recent retrospective real‐world analysis of US Flatiron Health records showed that, in addition to baseline anemia, the development of new or worsening anemia during the first 3 months of ruxolitinib treatment also had a significantly negative impact on survival.^18^ Nonetheless, the prognostic role of thrombocytopenia was not evaluated, and the clinical utility of the 12‐week time point for evaluating cytopenic trajectories remains uncertain, as it may be too early to guide meaningful clinical decisions. Furthermore, recent data have highlighted the prognostic significance of hemoglobin improvement during the first 24 weeks of momelotinib therapy, a JAK1/JAK2/activin receptor 1 (ACVR1) inhibitor, with improved overall survival (OS) observed in patients achieving an improvement in hemoglobin level.^19^

Given the complex interplay between MF biology, cytopenia development, and JAK inhibitor therapy, a more nuanced understanding of cytopenia trajectories during ruxolitinib treatment is needed. Specifically, it remains unclear how persistent versus treatment‐emergent cytopenia, different types of cytopenia (anemia vs. thrombocytopenia), and hemoglobin improvement in patients with baseline anemia affect prognosis and symptoms burden over a clinically relevant timeframe.

In this context, we conducted a subanalysis of the RUX‐MF study (NCT06516406), a large real‐world cohort of patients with MF treated with ruxolitinib, to provide a comprehensive evaluation of the prognostic impact of cytopenia, both persistent and treatment‐emergent, in 879 patients treated for at least 6 months.

Our objectives were to: (1) assess the prognostic impact of persistent and treatment‐emergent cytopenia during the first 6 months of therapy; (2) compare clinical outcomes between treatment‐emergent anemia and thrombocytopenia; (3) evaluate the prognostic significance of hemoglobin improvement in patients presenting with isolated baseline anemia; and (4) assess the impact of changes in cytopenia status on symptom burden, as measured by the Myeloproliferative Neoplasm Symptom Assessment Form Total Symptom Score (MPN‐SAF TSS).^20^

These findings aim to support risk stratification and guide therapeutic decision‐making in MF patients receiving ruxolitinib.

## MATERIALS AND METHODS

### Patient population and definitions

This subanalysis of the RUX‐MF study included 879 patients with myelofibrosis who received ruxolitinib for at least 6 months.

Diagnoses of primary myelofibrosis (PMF) and post‐polycythemia or essential thrombocythemia myelofibrosis (PPV/PET‐MF) were made according to 2016 World Health Organization criteria (WHO) and International Working Group on Myelofibrosis Research and Treatment (IWG‐MRT) criteria, respectively.^21^

The risk category was assessed at the time patients started on ruxolitinib according to the Dynamic International Prognostic Scoring System (DIPSS; for PMF) or myelofibrosis secondary to PV and ET‐prognostic model (MYSEC‐PM; for PPV‐ and PET‐MF).^5^, ^22^ Histologic examination was performed at local institutions; fibrosis was graded according to the European Consensus Grading System.^23^ High molecular risk (HMR) pathogenetic mutations were defined as those including *ASXL1*, *SRSF2*, *EZH2*, *IDH1* and *IDH2*, and *U2AF1* in next‐generation sequencing analysis.^24^, ^25^ Unfavorable karyotype was defined according to the DIPSS‐plus classification and included the presence of a complex karyotype or at least one of the following abnormalities: +8, −7/7q−, i(17q), −5/5q−, 12p−, inv(3), or 11q23 rearrangements.^26^ Anemia and thrombocytopenia were defined as hemoglobin (Hb) <10 g/dL and platelet count (PLT) <100 × 10^9^/L, respectively.^14^

MF‐related symptoms were assessed using the 10‐item MPN‐SAF TSS.^20^ Symptoms and spleen responses were defined according to 2013 IWG‐MRT criteria.^27^

Clinical and hematologic assessments were performed at baseline (ruxolitinib start) and during follow‐up as part of routine clinical management; all patients were followed according to standard practice procedures, regardless of enrollment in the study.

Patients were stratified into four groups: (1) no cytopenia at baseline and 6 months (“never cytopenic”); (2) no cytopenia at baseline but development of one or more cytopenia during follow‐up (“treatment‐emergent cytopenia”); (3) cytopenia present at both baseline and 6 months (“persistent cytopenia”); and (4) baseline anemia with Hb >10 g/dL at 6 months, in the absence of concurrent baseline or treatment‐emergent thrombocytopenia (“improved anemia”).

### Ethical aspects

The RUX‐MF study (NCT06516406) was performed in accordance with the guidelines of the institutional review boards of the participating centers and the standards of the Helsinki Declaration. The promoter of this study was the IRCCS Azienda Ospedaliero‐Universitaria S. Orsola‐Malpighi, Bologna, which obtained approval from the Area Vasta Emilia Centro Ethics Committee (approval file number: 048/2022/Oss/AOUBo). The study was approved by the local ethics committee of participating centers (protocol code: RUX‐MF) and has no commercial support.

### Statistical analysis

Statistical analysis was performed at the biostatistics laboratory of the myeloproliferative neoplasm unit at the Institute of Hematology “L. and A. Seràgnoli”, IRCCS Azienda Ospedaliero‐Universitaria di Bologna.

Descriptive statistics were used to summarize baseline characteristics. Continuous variables were expressed as mean ± standard deviation or median (range), as appropriate. Categorical variables were reported as frequencies and percentages. We used the Wilcoxon Mann‐Whitney rank sum test for comparisons between two groups, whereas Kruskal‐Wallis equality‐of‐populations rank test were used for comparison between three or more groups. Associations between categorical variables (2‐way tables) were tested using the Fisher exact test or the χ^2^ test, as appropriate.

Survival analyses were performed in patients who received ruxolitinib for at least 6 months. OS was estimated using the Kaplan–Meier method from the start of ruxolitinib therapy to death or last contact, whichever occurred first, and differences between groups were evaluated using the log‐rank test. Patients who underwent allogeneic stem cells transplantation were censored at the time of the procedure. This approach allowed the evaluation of cytopenia trajectories during the first 6 months of treatment while minimizing early discontinuation bias.

Hazard ratios (HRs) were estimated via Cox proportional hazards regression, along with their 95% confidence intervals (CIs). Multivariable Cox proportional hazards models were constructed to evaluate the independent association between cytopenia trajectory and overall survival, adjusting for clinically relevant baseline covariates that were unbalanced across groups, specifically: age ≥65 years, status of primary MF (vs. secondary MF), status of overt myelofibrosis (vs. pre‐fibrotic MF), bone marrow fibrosis ≥3, ruxolitinib starting dose, hemoglobin level <10 g/dL, leukocytes count <4 × 10^9^/L, platelets count <100 × 10^9^/L, peripheral blasts ≥1%, spleen length >10 cm below costal margin, and total symptoms score >20.

*p* values associated with HRs from pairwise Cox proportional hazards models evaluating overall survival, as well as *p* values derived from pairwise comparisons of symptom burden at baseline and symptom response at 6 months across cytopenia trajectory groups, were adjusted for multiple testing using the Benjamini–Hochberg false discovery rate procedure.

Tests were two‐sided, and *p* values <.05 were considered significant. Analyses were performed using STATA/SE software version 18.0 (StataCorp).

## RESULTS

### Patient characteristics and outcome according to baseline cytopenia

Among the 879 patients treated with ruxolitinib for at least 6 months, 357 patients (40.6%) presented with cytopenia at baseline, defined as Hb <10 g/dL and/or PLT <100 × 10^9^/L. Specifically, 301 patients (34.2%) had isolated anemia, 32 (3.6%) had isolated thrombocytopenia, and 24 (2.7%) had both at ruxolitinib start.

Patients with at least one baseline cytopenia were significantly older (mean age, 68.6 vs. 65.5 years, *p* < .001), had a higher prevalence of primary MF (58.5% vs. 46.9%, *p* = .001), higher DIPSS/MYSEC‐PM risk scores (intermediate‐2: 61.1% vs. 13.8%; high: 22.4% vs. 0%, *p* < .001), more elevated peripheral blood blast counts (mean, 1.2% vs. 0.8%, *p* < .001), and more severe symptom burden (mean TSS, 28.6 vs. 24.0, *p* < .001) compared with patients without cytopenia (Table S1). In particular, patients with both cytopenia (mean TSS: 28.2) and those with only anemia (mean TSS: 28.9) had a higher mean TSS compared to patients with only thrombocytopenia (mean TSS: 22.6, both *p* < .001) (Figure S1).

During a median follow‐up of 45.5 months (range, 6.0–181.2), 441 deaths occurred. Median OS for the entire cohort was 5.75 years (95% CI, 5.26–6.19).

Patients with at least one cytopenia at baseline had a significantly shorter median OS compared to those without (3.7 vs. 6.7 years; HR, 2.11; 95% CI, 1.75–2.54; *p* < .001) (Figure S2). No survival differences were detected between patients with anemia only, thrombocytopenia only and both cytopenia (median OS, 4.05, 3.85, and 4.41 years, respectively; log‐rank test *p* = .77).

### Outcome according to cytopenia trajectory

After 6 months of ruxolitinib therapy, the proportion of patients experiencing any form of cytopenia rose from 40.6% at baseline to 57.8% (*n* = 508).

**FIGURE 1:**
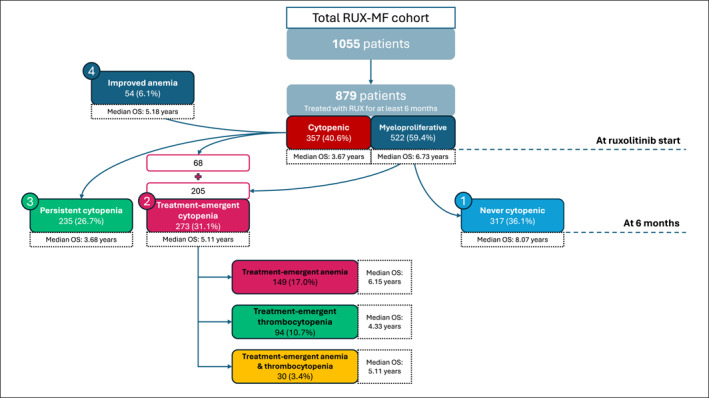
Flow diagram of patient allocation by cytopenia group and main results. Percentages were calculated on the cohort of patients treated with ruxolitinib (RUX) for at least 6 months (*n* = 879).

Based on the evolution of cytopenia during the first 6 months of treatment, patients were categorized into four distinct groups. Group 1, termed “never cytopenic,” comprised 317 patients (36.1%) who exhibited no cytopenia at baseline and at the 6‐month time point. Group 2, called “treatment‐emergent cytopenia,” included 273 patients (31.1%) who developed at least one new cytopenia during the first 6 months; of these, 205 were cytopenia‐free at baseline, whereas 68 developed an additional cytopenia. Group 3, labeled “persistent cytopenia,” consisted of 235 patients (26.7%) with cytopenia present both at baseline and at 6 months (24 patients with persistent both cytopenia; 191 with persistent anemia; 20 with persistent thrombocytopenia). Finally, group 4, referred to as “improved anemia,” encompassed 54 patients (6.1%) who had baseline anemia and achieved hemoglobin levels >10 g/dL at 6 months, without concurrent baseline or treatment‐emergent thrombocytopenia. Figure 1 provides a flow diagram of patient allocation and main outcomes.

Ruxolitinib doses at 6 months differed across the cytopenia trajectory groups (Table S1). Patients who remained never cytopenic were more likely to maintain higher ruxolitinib doses at 6 months (mean ruxolitinib dose: 28.7 mg/day ± SD: 9.5), whereas those with persistent (24.3 mg/day ± SD: 10.9) or treatment‐emergent (22.7 mg/day ± SD: 10.7) cytopenia more frequently required dose reductions (group 1: 32.2% vs. group 2: 40.3% and group 3: 36.2%). Table 1 resumed principal features according to the four groups defined above.

**TABLE 1:** Patients characteristics at ruxolitinib start based cytopenia evolution at 6 months.

	Never cytopenic (*n* = 317)	Treatment‐emergent cytopenia (*n* = 273)	Persistent cytopenia (*n* = 235)	Improved anemia (*n* = 54)	*p*
Age, mean (±SD), years	63.9 (10.9)	68.5 (10.0)	68.7 (9.0)	66.6 (11.1)	<.001
≥65 years, No. (%)	168 (53.0)	188 (68.9)	163 (69.4)	34 (63.0)	<.001
Male sex, No. (%)	187 (59.0)	150 (55.0)	127 (54.0)	32 (59.3)	.61
Primary myelofibrosis, No. (%)	143 (45.1)	148 (54.2)	134 (57.0)	27 (50.0)	.03
Overt myelofibrosis, No. (%)	139/205 (67.8)	149/183 (81.4)	127/153 (83.0)	29/37 (78.4)	.002
Bone marrow fibrosis ≥3, No. (%)	74 (23.3)	102 (37.4)	95 (40.4)	21 (38.9)	<.001
RUX starting dose, No. (%)					<.001
5 mg bid	19 (6.0)	45 (16.5)	53 (22.6)	7 (13.0)	
10 mg bid	72 (22.7)	63 (23.1)	41 (17.4)	17 (31.5)	
15 mg bid	61 (19.2)	72 (26.3)	60 (25.5)	12 (22.2)	
20 mg bid	165 (52.1)	93 (34.1)	81 (34.5)	18 (33.3)	
Driver mutation, No. (%)					.12
*JAK2* ^*V617F*^	257 (83.4)	209 (79.2)	159 (72.6)	39 (73.6)	
*CALR*	33 (10.7)	36 (13.6)	35 (16.0)	8 (15.0)	
*MPL*	5 (1.6)	8 (3.0)	6 (2.7)	3 (5.7)	
Triple‐negative	13 (4.2)	11 (4.2)	19 (8.7)	3 (5.7)	
Presence of HMR mutation, No. (%)	43/99 (43.4)	33/66 (50.0)	32/51 (62.8)	16/22 (72.7)	.03
Unfavorable karyotype, No. (%)	10/197 (5.1)	12/148 (8.1)	14/124 (11.3)	3/32 (8.6)	.24
Hemoglobin, mean (±SD), g/dL	13.0 (1.7)	11.2 (1.8)	9.1 (1.5)	9.5 (1.2)	<.001
<10 g/dL, No. (%)	0	52 (19.1)	207 (88.1)	54 (100.0)	<.001
Transfusion‐dependence, No. (%)	0	22 (8.1)	113 (48.1)	13 (24.1)	<.001
Leukocytes count, mean (±SD), ×10^9^/L	16.0 (11.0)	14.9 (12.6)	14.9 (15.8)	16.4 (14.8)	<.001
<4 × 10^9^/L, No. (%)^a^	12 (3.8)	23 (8.4)	30 (12.8)	7 (13.0)	.001
Platelets count, mean (±SD), ×10^9^/L	402 (231)	298 (231)	324 (262)	349 (259)	<.001
<100 × 10^9^/L, No. (%)	0	12 (4.4)	44 (18.7)	0	<.001
Peripheral blasts, mean (±SD), %	0.7 (1.2)	1.0 (1.8)	1.2 (1.7)	0.9 (1.4)	.002
≥1%, No. (%)	92 (29.0)	103 (37.7)	106 (45.1)	19 (35.2)	.003
Spleen length, mean (±SD), cm BCM	9.9 (6.3)	11.3 (6.6)	11.0 (6.3)	11.3 (6.9)	.02
>10 cm BCM, No. (%)	114 (36.0)	132 (48.4)	110 (46.8)	28 (51.9)	.006
TSS, mean (±SD)	23.0 (17.4)	26.8 (17.7)	27.1 (19.1)	35.3 (21.8)	<.001
>20, No. (%)	163 (51.4)	155 (56.8)	144 (61.3)	40 (74.1)	.001
DIPSS/MYSEC‐PM risk score, No. (%)					<.001
Intermediate‐1	277 (87.4)	190 (69.6)	39 (16.6)	11 (20.4)	
Intermediate‐2	40 (12.6)	71 (26.0)	142 (60.4)	35 (64.8)	
High	0	12 (4.4)	54 (23.0)	8 (14.8)	
Time from MF diagnosis to RUX start, mean (±SD), years	2.6 (4.5)	3.3 (4.6)	3.3 (4.9)	1.9 (2.4)	.01
>1 year, No. (%)	132 (41.6)	136 (49.8)	130 (55.3)	25 (46.3)	.01
>2 years, No. (%)	108 (34.1)	112 (41.0)	99 (42.1)	17 (31.5)	.12
Spleen response^b^, No. (%)	71/269 (26.4)	58/230 (25.2)	39/196 (19.9)	10/33 (30.3)	.33
Allogeneic stem cells transplant during follow‐up, No. (%)	30 (9.5)	26 (9.5)	23 (9.8)	6 (11.1)	.98

Abbreviations: BCM, below costal margin; bid, twice daily; DIPSS, Dynamic International Prognostic Scoring System; HMR, high molecular risk; MF, myelofibrosis; MYSEC‐PM, myelofibrosis secondary to PV and ET‐prognostic model; RUX, ruxolitinib; SD, standard deviation; TSS, total symptoms score.

^a^ No one with neutropenia.

^b^ Spleen response was defined according to International Working Group‐Myeloproliferative Neoplasms Research and Treatment criteria.

**FIGURE 2:**
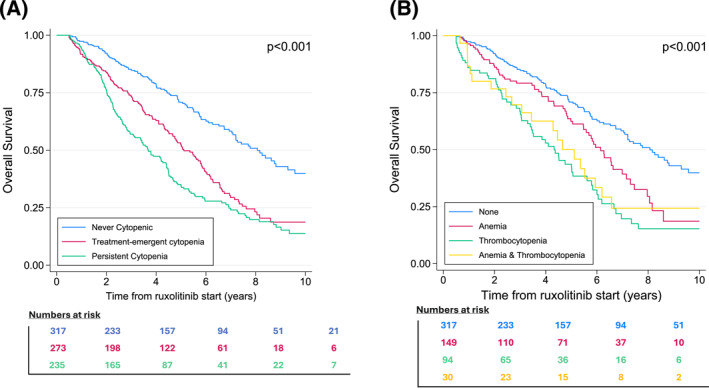
Kaplan–Meier curves for overall survival by (A) cytopenia trajectory and by (B) type of treatment‐emergent cytopenia.

The trajectory of cytopenia during the first 6 months of therapy had a significant impact on OS. Patients in the “never cytopenic” group had the longest survival, with a median OS of 8.07 years (95% CI, 7.04–9.29). In patients with treatment‐emergent cytopenia (group 2), the median OS was significantly lower: 5.11 years (95% CI, 4.66–5.84; HR, 2.01; 95% CI, 1.56–2.58; false discovery rate [FDR]‐adjusted *p* = .00003). Finally, patients in group 3 (“persistent cytopenia”) had the lowest survival, with a median OS of 3.68 years (95% CI, 3.08–4.41; HR, 2.62; 95% CI, 2.04–3.35; FDR‐adjusted *p* = .000008). Kaplan–Meier survival curves for each group are shown in Figure 2A. After adjustment by unbalanced characteristics, both treatment‐emergent and persistent cytopenia remained independently associated with inferior OS (*p* < .001) (Figure S3).

### Outcome according to the type of treatment‐emergent cytopenia

Within the treatment‐emergent cytopenia group (*n* = 273), we further analyzed outcomes based on the type of cytopenia developed during the first 6 months of ruxolitinib therapy.

Patients developing anemia alone (*n* = 149) had a median OS of 6.15 years (95% CI, 5.47–7.15), whereas those with isolated thrombocytopenia (*n* = 94) had a shorter median OS (4.33 years; 95% CI, 3.29–5.06; HR, 1.65; 95% CI, 1.15–2.34; FDR‐adjusted *p* = .008). Patients developing both cytopenia (*n* = 30) had a median OS of 5.11 years (95% CI, 2.67–6.20), comparable to patients with treatment‐emergent thrombocytopenia but significantly shorter than patients with treatment‐emergent anemia alone (HR, 1.43; 95% CI, 1.07–2.30, FDR‐adjusted *p* = .0114).

Compared with never cytopenia patients, treatment‐emergent anemia conferred an intermediate prognosis (HR, 1.63; 95% CI, 1.19–2.22; FDR‐adjusted *p* = .0032), whereas treatment‐emergent thrombocytopenia (HR, 2.51; 95% CI, 1.82–3.47; *p* < .001) and concurrent treatment‐emergent anemia and thrombocytopenia (HR, 2.17; 95% CI, 1.34–3.51; FDR‐adjusted *p* = .0032) were associated with the poorest outcomes (Figure 2B). This was confirmed after adjustment by unbalanced characteristics (*p* < .001) (Figure S4).

### Outcome according to hemoglobin improvement

**FIGURE 3:**
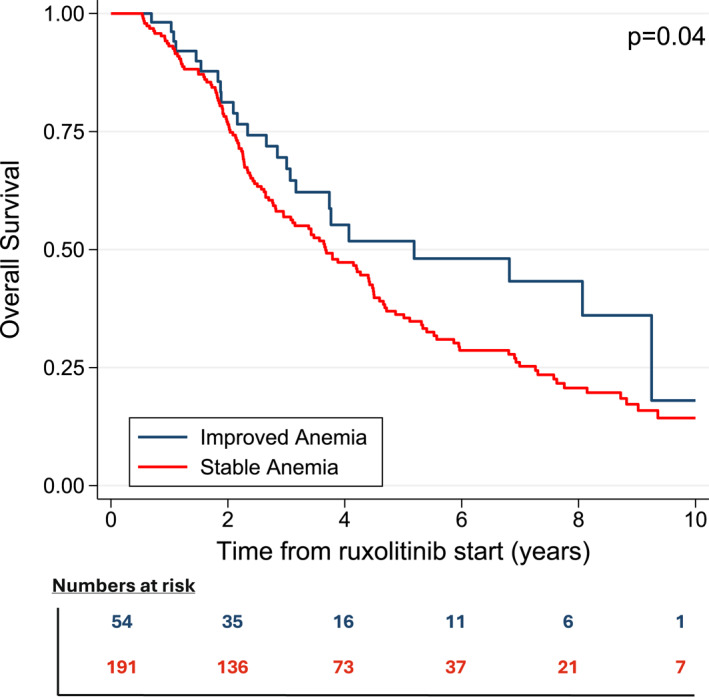
Overall survival by hemoglobin improvement at 6 months.

Overall, 301 patients presented baseline anemia only. Among these, 54 (17.9%) patients achieved Hb >10 g/dL at 6 months without treatment‐emergent thrombocytopenia (“improved anemia” group). These patients had a median OS of 5.18 years (95% CI, 3.07–9.25), significantly longer compared to 3.47 years (95% CI, 2.95–4.42) for those with persistent anemia (HR, 1.46; 95% CI, 1.01–2.12; FDR‐adjusted *p* = .043) (Figure 3).

### Correlation between cytopenia and symptoms burden

The evolution of cytopenia during the first 6 months of ruxolitinib therapy was associated with significant changes in symptom burden.

At baseline, group 4 showed the higher symptoms burden (mean MPN‐SAF TSS: 35.3, SD: 21.8), compared to the other three groups (group 3: 27.1, FDR‐adjusted *p* = .005; group 2: 26.8, FDR‐adjusted *p* = .006; and group 1: 23.0, FDR‐adjusted *p* = .003).

**FIGURE 4:**
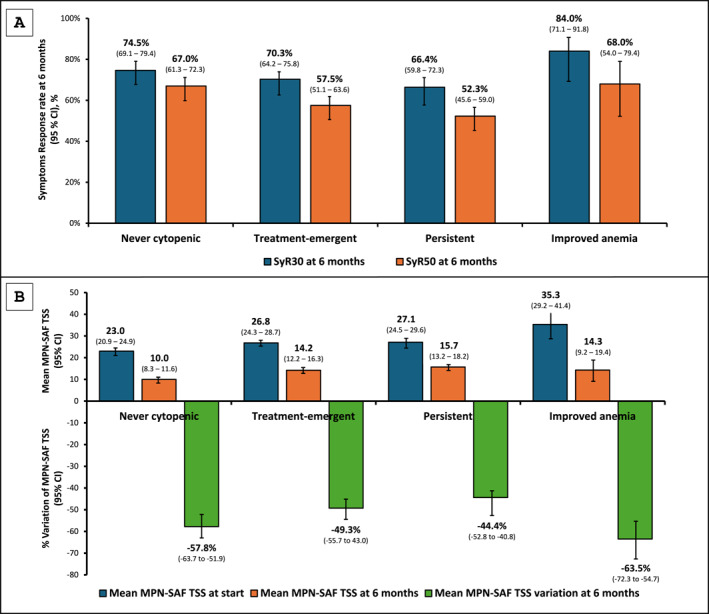
(A) Symptoms’ response at 6 months and (B) mean MPN‐SAF TSS and percentage variation between baseline and 6 months after ruxolitinib across the four groups. SyR30 symptoms’ response rate defined as a reduction of at least 30% respect TSS at baseline; SyR50, symptoms’ response rate defined as a reduction of at least 50% respect TSS at baseline. CI indicates confidence interval; MPN‐SAF, myeloproliferative neoplasm symptom assessment form; TSS, total symptom score.

During the first 6 months of ruxolitinib treatment, group 1 and group 4 showed higher symptom response rates (67.0% and 68.0%, respectively). After adjustment for multiple testing, a significantly higher response rate was confirmed for group 1 compared with group 3 (FDR‐adjusted *p* = .004), whereas differences involving groups 2 and 4 did not retain statistical significance (Figure 4A; Table S4). Consistent with these responses, groups 4 and 1 exhibited the highest mean percentage decrease in MPN‐SAF TSS, with mean reductions of –63.5% and –57.8%, respectively. Groups 2 and 3 showed lower mean reductions of –49.3% and –44.4%, respectively (Figure 4B).

In addition, the improved anemia group showed a statistically significant lower symptoms burden (mean TSS: 14.3, SD: 19.9) compared directly with patients with persistent anemia at 6 months (mean TSS: 16.4, SD: 19.6, *p* = .03).

## DISCUSSION

In this cohort, the prevalence of cytopenia rose markedly during ruxolitinib treatment, from 40.6% at baseline to 57.8% at 6 months. This notable increase reflects, on one hand, the myelosuppressive properties of ruxolitinib and, on the other hand, may serve as an indicator of limited marrow reserve and/or biologically more aggressive disease, predisposing to early hematologic toxicity.

Crucially, both persistent and treatment‐emergent cytopenia during the first 6 months of therapy were independently associated with a doubling of the risk of death compared to patients who remained cytopenia‐free.

This observation aligns with prior evidence indicating that anemia at ruxolitinib initiation, and its early worsening within the first 3 months, predicts poorer clinical outcomes.^18^, ^28^, ^29^, ^30^ Our study extends these findings by demonstrating that not only baseline cytopenia, but also the development of new cytopenia over a longer treatment window of 6 months, holds substantial prognostic relevance. The 6‐month mark represents a more clinically actionable time point, enabling appropriate dose optimization and implementation of supportive measures, while minimizing premature treatment discontinuation or undue concern. These results underscore the evolving nature of hematologic risk during ruxolitinib therapy and reinforce the importance of continued blood count surveillance.

Among the different cytopenic profiles, treatment‐emergent thrombocytopenia, either isolated or in combination with anemia, was identified as the most adverse prognostic feature. This observation is particularly relevant, as thrombocytopenia has been less emphasized than anemia in prior prognostic models and treatment goals. Our results suggest that platelet count trajectories during ruxolitinib therapy should be closely monitored and considered in risk stratification, potentially informing the need for alternative therapies.

Interestingly, recovery from anemia without concurrent thrombocytopenia was uncommon but was associated with improved survival compared to patients with persistent anemia. Nonetheless, overall survival in this subgroup was comparable to that of patients who developed treatment‐emergent cytopenia, indicating that baseline anemia retains its adverse prognostic significance even when hematologic improvement is achieved. These findings suggest that a hemoglobin improvement, although beneficial, may reflect partial disease control rather than complete mitigation of the underlying adverse biology. Our results are consistent with recent findings from the SIMPLIFY‐1 and SIMPLIFY‐2 study, where achieving Hb >10 g/dL with momelotinib at 6 months was associated with improved survival.^31^

Beyond its association with improved survival, the recovery of hemoglobin levels during ruxolitinib therapy also appeared to parallel a meaningful amelioration of symptom burden. Patients achieving hemoglobin improvement, despite entering treatment with the most severe baseline symptoms, experienced a substantially higher rate of symptom response at 6 months (68.0%) compared with those with persistent (52.3%) or treatment‐emergent cytopenia (57.5%). This observation suggests that erythroid recovery may not only reflect improved marrow function but also translate into a tangible enhancement in patient well‐being. From a clinical perspective, these findings emphasize how correcting anemia can improve patients' overall quality of life, a dimension that is often profoundly impaired in myelofibrosis.^32^, ^33^ The parallel improvement in hematologic and symptomatic domains highlights the multidimensional benefit of effective JAK inhibition, reinforcing the importance of monitoring patient‐reported outcomes as complementary measures of treatment efficacy.^34^, ^35^

Furthermore, patients who remained cytopenia‐free throughout the first 6 months of therapy had the most favorable prognosis, with a median OS exceeding 8 years. This finding identifies a subset of MF patients who derive the greatest benefit from ruxolitinib and may guide positive expectations for long‐term outcomes. Conversely, baseline cytopenia, as well as the early development or persistence of cytopenia, may serve as an early warning sign of suboptimal response or disease progression, warranting consideration of alternative front‐line therapeutic strategies and closer surveillance.

We acknowledge several limitations of this study, including its retrospective design, the variable quality and completeness of data, and the absence of standardized patient management across participating centers, all of which may have influenced survival outcomes. In particular, the use of erythropoiesis‐stimulating agents (ESAs) was not standardized and likely varied according to physician experience and local policies. For this reason, the use of ESAs was not included in multivariable models, as information on timing and duration of ESA exposure was not uniformly available, precluding a reliable time‐dependent analysis. A further limitation of this study is the incomplete availability of cytogenetic and molecular data. This is in line with real‐world clinical workflows, which implement next‐generation sequencing and comprehensive cytogenetic assessment only in a subset of cases. Future studies, ideally with a prospective design, will be needed to integrate detailed molecular, cytogenetic, and supportive care data, including the timing and duration of erythropoiesis‐stimulating agent use.

Nonetheless, these findings extend and refine current understanding of cytopenia dynamics during ruxolitinib therapy and their implications for patient outcomes, symptoms burden, and quality of life.

## AUTHOR CONTRIBUTIONS

**Francesca Palandri**: Conceptualization, visualization, investigation, data curation, funding acquisition, resources, writing–original draft, and writing–review and editing. **Giovanni Caocci**: Conceptualization, visualization, investigation, resources, and writing–review and editing. **Elisabetta Abruzzese**: Conceptualization, visualization, investigation, resources, and writing–review and editing. **Mario Tiribelli**: Conceptualization, visualization, investigation, resources, and writing–review and editing. **Erika Morsia**: Conceptualization, visualization, investigation, resources, and writing–review and editing. **Mirko Farina**: Conceptualization, visualization, investigation, resources, and writing–review and editing. **Giulia Benevolo**: Conceptualization, visualization, investigation, resources, and writing–review and editing. **Eloise Beggiato**: Investigation, resources, and writing–review and editing. **Bruno Martino**: Investigation, resources, and writing–review and editing. **Novella Pugliese**: Investigation, resources, and writing–review and editing. **Alessia Tieghi**: Investigation, resources, and writing–review and editing. **Monica Crugnola**: Investigation, resources, and writing–review and editing. **Gianni Binotto**: Investigation, resources, and writing–review and editing. **Francesco Cavazzini**: Investigation, resources, and writing–review and editing. **Alessandra Iurlo**: Investigation, resources, and writing–review and editing. **Alessandro Isidori**: Investigation, resources, and writing–review and editing. **Alessandra Dedola**: Investigation, resources, and writing–review and editing. **Emilia Scalzulli**: Investigation, resources, and writing–review and editing. **Andrea Duminuco**: Investigation, resources, and writing–review and editing. **Daniele Cattaneo**: Investigation, resources, and writing–review and editing. **Roberto M. Lemoli**: Investigation, resources, and writing–review and editing. **Costanza Bosi**: Investigation, resources, and writing–review and editing. **Daniela Cilloni**: Investigation, resources, and writing–review and editing. **Monica Bocchia**: Investigation, resources, and writing–review and editing. **Fabrizio Pane**: Investigation, resources, and writing–review and editing. **Chiara Sartor**: Investigation, resources, and writing–review and editing. **Florian H. Heidel**: Visualization and writing–review and editing. **Massimo Breccia**: Conceptualization, visualization, investigation, resources, and writing–review and editing. **Filippo Branzanti**: Conceptualization, visualization, data curation, formal analysis, writing–original draft, and writing–review and editing. **Giuseppe A. Palumbo**: Conceptualization, visualization, investigation, resources, and writing–review and editing. **Massimiliano Bonifacio**: Conceptualization, investigation, visualization, resources, and writing–review and editing. **Elena M. Elli**: Conceptualization, investigation, visualization, resources, and writing–review and editing.

## CONFLICT OF INTEREST STATEMENT

Francesca Palandri reports honoraria and consulting fees from Novartis, GSK, BMS, Incyte, Sanofi, Takeda, Sobi, and AOP. Elisabetta Abruzzese reports honoraria and consulting fees from Novartis, GSK, BMS, Incyte, Ascentage, and Pfizer. Mario Tiribelli reports honoraria from and has served on speakers’ bureaus for Novartis, BMS, Pfizer, and Incyte. Monica Crugnola reports honoraria from Novartis and Amgen. Gianni Binotto reports honoraria from Novartis, Incyte, BMS‐Celgene, and Pfizer. Roberto M. Lemoli reports honoraria from Jazz, Pfizer, AbbVie, BMS, Sanofi, and StemLine. Fabrizio Pane reports honoraria from Incyte, Novartis, Jazz, BMS‐Celgene, Amgen, and Gilead. Florian H. Heidel reports consulting fees from BMS/Celgene, Novartis, CTI, AOP, Janssen, GSK, MSD, AbbVie, Takeda, Kartos, Geron, Silence, Prelude, and Sumitomo; and research funding from BMS/Celgene and Novartis. Massimo Breccia reports honoraria from Novartis, BMS, Pfizer, and Incyte. Giuseppe A. Palumbo reports consultancy and honoraria from AbbVie, AOP, AstraZeneca, BMS, Incyte, GSK, Morphosys, and Novartis. Massimiliano Bonifacio reports honoraria from Novartis, BMS, Pfizer, and Incyte. Elena M. Elli reports meeting/advisory board participation for Novartis, GSK, AbbVie, and AOP. Giulia Benevolo reports consulting fees from Bristol‐Myers Squibb, GlaxoSmithKline, and Novartis; and fees for other professional activities from AOP Health and Janssen Biotech. Monica Bocchia reports consulting fees from Incyte Corporation and Novartis; and fees for travel from BeiGene USA, Inc. Daniela Cilloni reports fees for professional activities from AbbVie, Celgene Corporation, Jazz Pharmaceuticals, and Novartis Pharma; and travel fees from Bristol‐Myers Squibb. Andrea Duminuco reports consulting fees from A.O.U. Policlinico “G.Rodolico‐San Marco”.

## Supporting information

Supporting Information S1

## Data Availability

The data that support the findings of this study are available from the corresponding author on reasonable request to the corresponding author (filippo.branzanti2@unibo.it) at the following DOI: 10.5281/zenodo.17462804.

## References

[1] Passamonti F , Mora B . Myelofibrosis. Blood. 2023;141(16):1954‐1970. doi:10.1182/BLOOD.2022017423 36416738 PMC10646775

[2] Passamonti F , Harrison CN , Mesa RA , Kiladjian JJ , Vannucchi AM , Verstovsek S . Anemia in myelofibrosis: current and emerging treatment options. Crit Rev Oncol Hematol. 2022;180:103862. doi:10.1016/J.CRITREVONC.2022.103862 36332787

[3] Verstovsek S . How I manage anemia related to myelofibrosis and its treatment regimens. Ann Hematol. 2023;102(4):689‐698. doi:10.1007/S00277-023-05126-4 36786879 PMC9998582

[4] Naymagon L , Mascarenhas J . Myelofibrosis‐related anemia: current and emerging therapeutic strategies. HemaSphere. 2017;1(1):e1. doi:10.1097/HS9.0000000000000001 31723730 PMC6745971

[5] Passamonti F , Cervantes F , Vannucchi AM , et al. A dynamic prognostic model to predict survival in primary myelofibrosis: a study by the IWG‐MRT (International Working Group for Myeloproliferative Neoplasms Research and Treatment). Blood. 2010;115(9):1703‐1708. doi:10.1182/BLOOD-2009-09-245837 20008785

[6] Tefferi A , Lasho TL , Jimma T , et al. One thousand patients with primary myelofibrosis: the mayo clinic experience. Mayo Clin Proc. 2012;87(1):25‐33. doi:10.1016/J.MAYOCP.2011.11.001 22212965 PMC3538387

[7] Mora B , Maffioli M , Rumi E , et al. Incidence of blast phase in myelofibrosis according to anemia severity. EJHaem. 2023;4(3):679‐689. doi:10.1002/JHA2.745 37601878 PMC10435699

[8] Nicolosi M , Mudireddy M , Lasho TL , et al. Sex and degree of severity influence the prognostic impact of anemia in primary myelofibrosis: Analysis based on 1109 consecutive patients. Leukemia. 2018;32(5):1254‐1258. doi:10.1038/S41375-018-0028-X 29568091 PMC5940639

[9] Elena C , Passamonti F , Rumi E , et al. Red blood cell transfusion‐dependency implies a poor survival in primary myelofibrosis irrespective of IPSS and DIPSS. Haematologica. 2011;96(1):167‐170. doi:10.3324/HAEMATOL.2010.031831 20884708 PMC3012782

[10] Mascarenhas J , Hoffman R . Ruxolitinib: the first FDA approved therapy for the treatment of myelofibrosis. Clin Cancer Res. 2012;18(11):3008‐3014. doi:10.1158/1078-0432.CCR-11-3145 22474318

[11] Harrison CN , Schaap N , Mesa RA . Management of myelofibrosis after ruxolitinib failure. Ann Hematol. 2020;99(6):1177‐1191. doi:10.1007/S00277-020-04002-9 32198525 PMC7237516

[12] Palandri F , Palumbo GA , Benevolo G , et al. Incidence of blast phase in myelofibrosis patients according to anemia severity at ruxolitinib start and during therapy. Cancer. 2024;130(8):1270‐1280. doi:10.1002/CNCR.35156 38153814

[13] Maffioli M , Mora B , Iurlo A , et al. The 2024 three‐strata baseline anemia definition of the revised IWG‐ELN criteria dissects survival in ruxolitinib‐treated myelofibrosis patients. Am J Hematol. 2025;100(9):1656‐1659. doi:10.1002/AJH.27734 40478635

[14] Palandri F , Breccia M , Mazzoni C , et al. Ruxolitinib in cytopenic myelofibrosis: response, toxicity, drug discontinuation, and outcome. Cancer. 2023;129(11):1704‐1713. doi:10.1002/CNCR.34722 36932983

[15] Palandri F , Breccia M , Morsia E , et al. Disease phenotype significantly influences the outcome after discontinuation of ruxolitinib in chronic phase myelofibrosis. Clin Lymphoma Myeloma Leuk. 2025;25(7):e524‐e532.e3. doi:10.1016/j.clml.2025.02.015 40133140

[16] Maffioli M , Mora B , Ball S , et al. A prognostic model to predict survival after 6 months of ruxolitinib in patients with myelofibrosis. Blood Adv. 2022;6(6):1855‐1864. doi:10.1182/BLOODADVANCES.2021006889 35130339 PMC8941454

[17] Palandri F , Branzanti F , Bonifacio M , et al. Revised “iRR6” model in intermediate‐1 risk myelofibrosis patients treated with ruxolitinib. Cancer. 2025;131(17):e70062. doi:10.1002/CNCR.70062 40839414 PMC12369922

[18] Kuykendall AT , Palandri F , Zhang S , et al. Retrospective real‐world analysis of survival outcomes in patients with myelofibrosis and new or worsening anemia treated with ruxolitinib in the United States. Blood. 2024;144(suppl 1):3804. doi:10.1182/BLOOD-2024-205380

[19] Palandri F , Casey O’C , Pankit V , et al. Survival impact and kinetics of hemoglobin improvement with myelofibrosis and moderate to severe anemia: post hoc analyses of SIMPLIFY‐1 and MOMENTUM. Accessed October 8, 2025. https://library.ehaweb.org/eha/2025/eha2025‐congress/4160235/francesca.palandri.survival.impact.and.kinetics.of.hemoglobin.improvement.with.html?f=

[20] Emanuel RM , Dueck AC , Geyer HL , et al. Myeloproliferative neoplasm (MPN) symptom assessment form total symptom score: prospective international assessment of an abbreviated symptom burden scoring system among patients with MPNs. J Clin Oncol. 2012;30(33):4098‐4103. doi:10.1200/JCO.2012.42.3863 23071245 PMC4872304

[21] Arber DA , Orazi A , Hasserjian R , et al. The 2016 revision to the World Health Organization classification of myeloid neoplasms and acute leukemia. Blood. 2016;127(20):2391‐2405. doi:10.1182/BLOOD-2016-03-643544 27069254

[22] Passamonti F , Giorgino T , Mora B , et al. A clinical‐molecular prognostic model to predict survival in patients with post polycythemia vera and post essential thrombocythemia myelofibrosis. Leukemia. 2017;31(12):2726‐2731. doi:10.1038/leu.2017.169 28561069

[23] Gianelli U , Vener C , Bossi A , et al. The European consensus on grading of bone marrow fibrosis allows a better prognostication of patients with primary myelofibrosis. Mod Pathol. 2012;25(9):1193‐1202. doi:10.1038/modpathol.2012.87 22627739

[24] Tefferi A , Guglielmelli P , Lasho TL , et al. MIPSS70+ version 2.0: mutation and karyotype‐enhanced international prognostic scoring system for primary myelofibrosis. J Clin Oncol. 2018;36(17):1769‐1770. doi:10.1200/JCO.2018.78.9867 29708808

[25] Vannucchi AM , Lasho TL , Guglielmelli P , et al. Mutations and prognosis in primary myelofibrosis. Leukemia. 2013;27(9):1861‐1869. doi:10.1038/leu.2013.119 23619563

[26] Gangat N , Caramazza D , Vaidya R , et al. DIPSS plus: a refined Dynamic International Prognostic Scoring System for primary myelofibrosis that incorporates prognostic information from karyotype, platelet count, and transfusion status. J Clin Oncol. 2011;29(4):392‐397. doi:10.1200/JCO.2010.32.2446 21149668

[27] Tefferi A , Cervantes F , Mesa R , et al. Revised response criteria for myelofibrosis: International Working Group‐Myeloproliferative Neoplasms Research and Treatment (IWG‐MRT) and European LeukemiaNet (ELN) consensus report. Blood. 2013;122(8):1395‐1398. doi:10.1182/BLOOD-2013-03-488098 23838352 PMC4828070

[28] Laganà A , Scalzulli E , Carmosino I , et al. Baseline, drug‐related and persistent anemia and/or thrombocytopenia predict responses and prognosis in myelofibrosis patients treated with ruxolitinib. Hematol Oncol. 2025;43(3). doi:10.1002/HON.70086 40256999

[29] Gupta V , Harrison C , Hexner EO , et al. The impact of anemia on overall survival in patients with myelofibrosis treated with ruxolitinib in the COMFORT studies. Haematologica. 2016;101(12):e482‐e484. doi:10.3324/HAEMATOL.2016.151449 27587385 PMC5479619

[30] Gupta V , Guglielmelli P , Hamer‐Maansson J , Braunstein EM , Al‐Ali HK . Effect of new or worsening anemia on clinical outcomes in 2233 patients with myelofibrosis treated with ruxolitinib in the expanded‐access JUMP study. Blood. 2023;142(suppl 1):5174. doi:10.1182/BLOOD-2023-179417

[31] Mesa R , Harrison C , Oh ST , et al. Overall survival in the SIMPLIFY‐1 and SIMPLIFY‐2 phase 3 trials of momelotinib in patients with myelofibrosis. Leukemia. 2022;36(9):2261‐2268. doi:10.1038/S41375-022-01637-7;SUBJMETA 35869266 PMC9417985

[32] Mesa RA , Harrison C , Palmer JM , et al. Patient‐reported outcomes and quality of life in anemic and symptomatic patients with myelofibrosis: results from the MOMENTUM study. HemaSphere. 2023;7(11):E966. doi:10.1097/HS9.0000000000000966 37901848 PMC10599984

[33] LeBlanc TW , Collacott H , García Gutiérrez V , et al. Experienced or perceived burdens and associated quality of life impacts of anemia and transfusion dependence in myelofibrosis: a patient self‐report survey analysis. Blood. 2024;144(Supplement 1):3815. doi:10.1182/BLOOD-2024-206086

[34] Palandri F , Auteri G , Abruzzese E , et al. Ruxolitinib adherence in myelofibrosis and polycythemia vera: the “RAMP” Italian multicenter prospective study. Ann Hematol. 2024;103(6):1931‐1940. doi:10.1007/S00277-024-05704-0 38478023 PMC11090921

[35] Harrison CN , Mesa RA , Kiladjian JJ , et al. Health‐related quality of life and symptoms in patients with myelofibrosis treated with ruxolitinib versus best available therapy. Br J Haematol. 2013;162(2):229‐239. doi:10.1111/BJH.12375 23672349

