# Supplementary material for: Survival and quality‐of‐life implications of cytopenia trajectories in ruxolitinib‐treated myelofibrosis
Source: Cancer. 2026 Feb 13;132(4):e70320. doi: 10.1002/cncr.70320 (PMC12904337; doi:10.1002/cncr.70320)
Supplement: Supplementary file 1 — Supporting Information S1 [file CNCR-132-e70320-s001.pdf]

Supplemental Table 1: Baseline patient characteristics by cytopenia status

	Total (n. 879)	Baseline cytopenia (n. 357)	No baseline cytopenia (n. 522)	p-value
Age, mean (±SD), years	66.8 (10.4)	68.6 (9.7)	65.5 (10.7)	**<0.001**
≥65 years, n. (%)	554 (63.0)	247 (69.2)	307 (58.8)	**0.002**
Male sex, n. (%)	497 (56.5)	195 (54.6)	302 (57.9)	0.33
Primary Myelofibrosis, n. (%)	454 (51.6)	209 (58.5)	245 (46.9)	**0.001**
Overt Myelofibrosis, n. (%)	459/597 (76.9)	215/258 (83.3)	244/339 (72.0)	**0.001**
Bone marrow fibrosis ≥3, n. (%)	294 (33.5)	151 (42.3)	143 (27.4)	**<0.001**
RUX starting dose, n. (%)				**<0.001**
5 mg BID	128 (14.6)	85 (23.8)	43 (8.2)	
10 mg BID	195 (22.2)	76 (21.3)	119 (22.8)	
15 mg BID	205 (23.3)	91 (25.5)	114 (21.8)	
20 mg BID	351 (39.9)	105 (29.4)	246 (47.1)	
Driver mutation, n. (%)				**0.015**
JAK2^{V617F}	692 (78.7)	263 (73.7)	429 (82.1)	
CALR	117 (13.3)	55 (15.4)	62 (11.8)	
MPL	24 (2.7)	16 (4.5)	8 (1.6)	
Triple-Negative	47 (5.4)	23 (6.4)	24 (4.5)	
Presence of HMR mutation, n. (%)	116/242 (47.9)	57/90 (63.3)	69/152 (45.4)	**0.007**
Hemoglobin, mean (±SD), g/dL	11.2 (2.3)	9.3 (1.5)	12.6 (1.7)	**<0.001**
<10 g/dL, n. (%)	325 (37.0)	325 (91.0)	0	**<0.001**
Transfusion-dependence, n. (%)	151 (17.2)	151 (42.3)	0	**<0.001**
Leukocytes count, mean (±SD), ×10^9/L	15.5 (13.9)	15.0 (16.5)	15.8 (11.6)	**<0.001**
<4 x10^9/L, n. (%)	72 (8.2)	47 (13.2)	25 (4.8)	**<0.001**
Platelets count, mean (±SD), ×10^9/L	340.4 (245.4)	289.2 (244.5)	378.3 (239.3)	**<0.001**
<100 x10^9/L, n. (%)	56 (6.4)	56 (15.7)	0	**<0.001**
Peripheral blasts, mean (±SD), %	1.0 (1.6)	1.2 (1.7)	0.8 (1.5)	**<0.001**
≥1%, n. (%)	325 (37.0)	157 (44.0)	168 (32.2)	**0.001**
Spleen length, mean (±SD), cm BCM	10.8 (6.5)	11.6 (6.5)	10.3 (6.4)	**0.02**
>10 cm BCM, n. (%)	387 (44.0)	183 (51.3)	204 (39.1)	**0.001**
Total Symptoms Score, mean (±SD)	26.0 (18.5)	28.6 (19.3)	24.0 (17.6)	**<0.001**
>20, n. (%)	499/821 (60.8)	229/326 (70.2)	270/495 (54.6)	**<0.001**
DIPSS/MYSEC-PM risk score, n. (%)				**<0.001**
Intermediate-1	509 (57.9)	59 (16.5)	450 (86.2)	
Intermediate-2	290 (33.0)	218 (61.1)	72 (13.8)	
High	80 (9.1)	80 (22.4)	0	
Time from MF diagnosis to RUX start, mean (±SD), years	3.0 (4.6)	3.3 (4.8)	2.8 (4.4)	**0.001**
>1 year, n. (%)	425 (48.3)	199 (55.7)	226 (43.3)	**<0.001**
>2 years, n. (%)	337 (38.3)	152 (42.6)	185 (35.4)	**0.036**
Spleen Response†, n. (%)	178/728 (24.5)	64/288 (22.2)	114/440 (25.9)	0.26
Allogeneic stem cells transplant during follow-up, n. (%)	87 (9.9%)	45 (8.6)	42 (11.8)	0.13

*Supplemental Table 1: *no one with neutropenia. †Spleen response was defined according to IWG-MRT criteria. SD, standard deviation; BID, bis in die; BCM, below costal margin; MF, myelofibrosis; RUX, ruxolitinib; HMR, high molecular risk*

Supplemental Table 2: Dose modification at 6 months

Group	Characteristics	At RUX start	At 6 months	With Increased Continuous Values	With Decreased Continuous Values	With Stable Values
**Never Cytopenic (n. 317)**	Ruxolitinib dose, mean (±SD), mg/die	31.7 (9.8)	28.7 (9.5)	42 (13.2%)	102 (32.2%)	173 (54.6%)
**Treatment-emergent cytopenia (n. 273)**	Ruxolitinib dose, mean (±SD), mg/die	27.8 (10.9)	22.7 (10.7)	25 (9.2%)	110 (40.3%)	138 (50.5%)
**Persistent cytopenia (n. 235)**	Ruxolitinib dose, mean (±SD), mg/die	27.2 (11.6)	24. 3 (10.9)	38 (16.2%)	85 (36.2%)	112 (47.6%)
**Improved anemia (n. 54)**	Ruxolitinib dose, mean (±SD), mg/die	28.1 (10.3)	27.0 (10.3)	12 (22.2%)	10 (18.5%)	32 (59.3%)

Supplemental Table 3: Unadjusted and false discovery rate–adjusted p values from pairwise comparisons of overall survival across cytopenia trajectory groups.

Reference Group	Compared Group	Unadjusted p-value	FDR-adjusted p-value (BH)*
Never cytopenic	Persistent cytopenia	0.000001	0.000008
Never cytopenic	Treatment-emergent cytopenia	0.00001	0.0000267
Never cytopenic	Treatment-emergent thrombocytopenia	0.00001	0.0000267
Never cytopenic	Treatment-emergent anemia and thrombocytopenia	0.002	0.0032
Never cytopenic	Treatment-emergent anemia	0.002	0.0032
Treatment-emergent anemia	Treatment-emergent thrombocytopenia	0.006	0.008
Treatment-emergent anemia	Treatment-emergent anemia and thrombocytopenia	0.010	0.0114
Improved anemia	Persistent anemia	0.043	0.043

*Supplemental Table 3: *adjusted for multiple testing using the Benjamini–Hochberg false discovery rate procedure*

Supplemental Table 4: Unadjusted and false discovery rate–adjusted p-values from pairwise comparisons of baseline symptoms burden and symptoms response at 6 months across cytopenia trajectory groups.

	**Compared Groups**	**Unadjusted *p*-value**	**FDR-adjusted *p*-value (BH)***
**Baseline Symptoms Burden**	Group 4 vs Group 1	<0.001	0.003
	Group 4 vs Group 2	0.004	0.006
	Group 4 vs Group 3	0.005	0.005
**Symptoms Response at 6 months**	Group 1 vs Group 3	0.001	0.004
	Group 1 vs Group 2	0.03	0.06
	Group 4 vs Group 3	0.04	0.053
	Group 4 vs Group 2	0.05	0.05

*Supplemental Table 4: *adjusted for multiple testing using the Benjamini–Hochberg false discovery rate procedure*

Supplemental Figure 1: Symptoms burden at ruxolitinib start based on cytopenia status

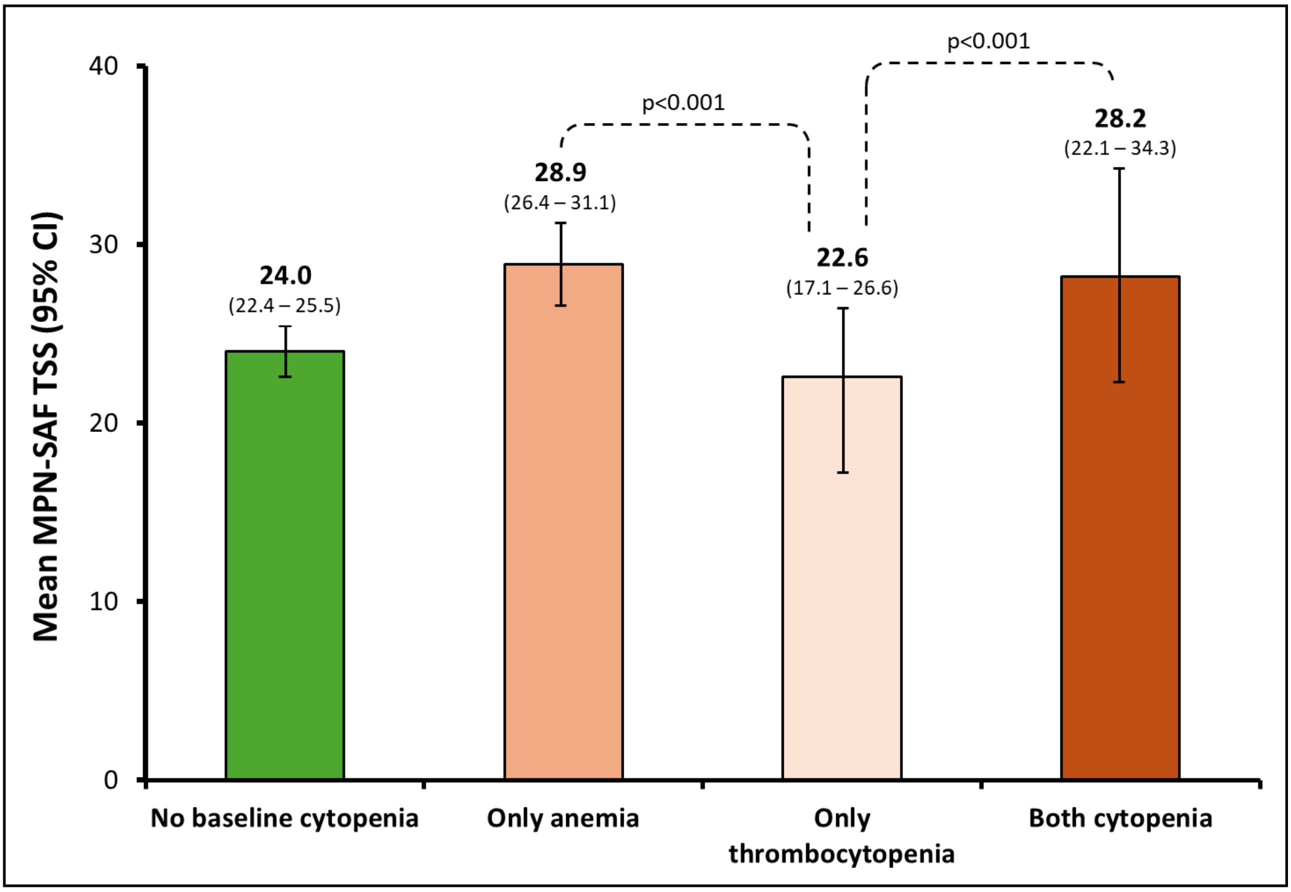

Supplemental Figure 1: MPN-SAF TSS, myeloproliferative neoplasm symptoms assessment form total symptoms score; CI, confidence interval.

Supplemental Figure 2: Kaplan-Meier curves for OS by baseline cytopenia status

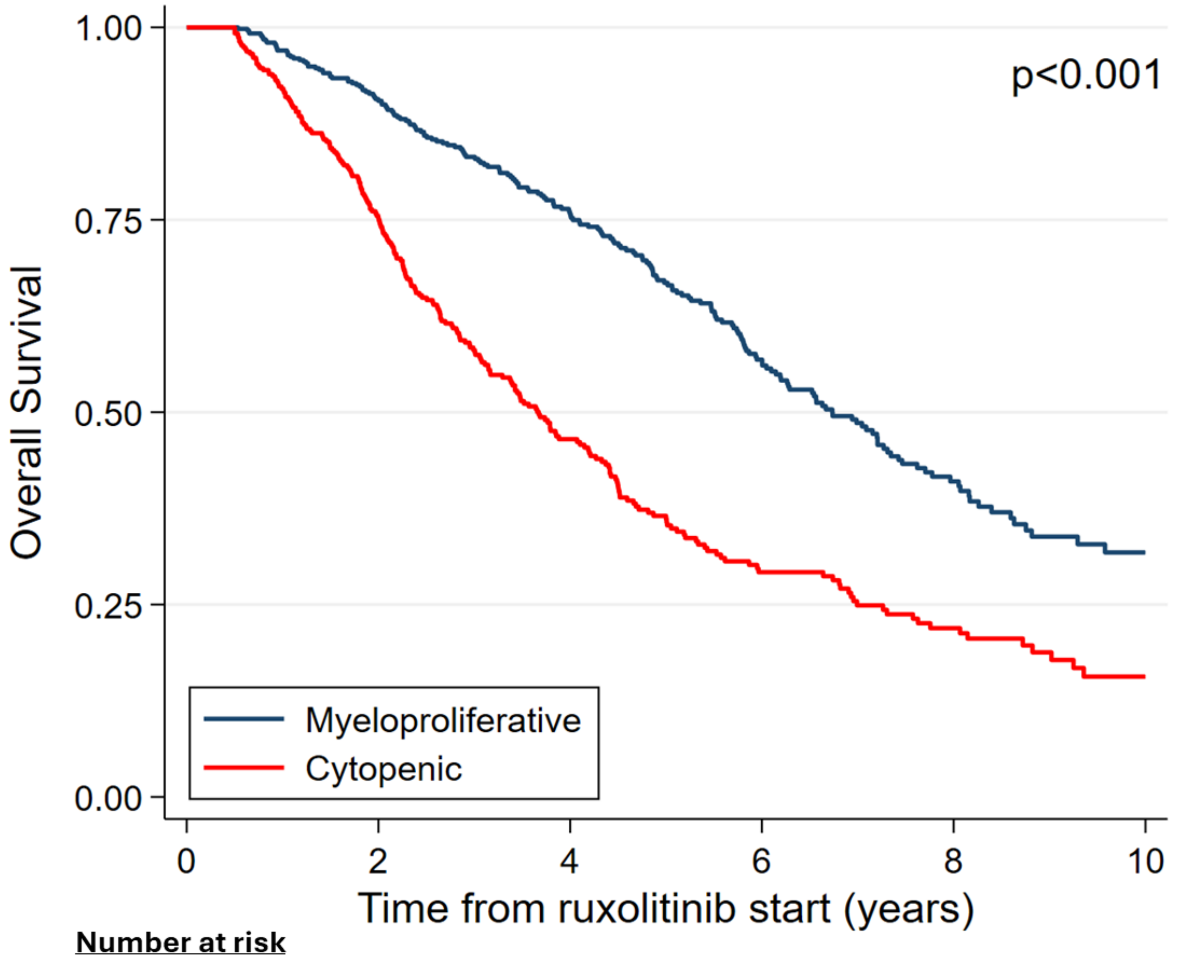

Number at risk

522	388	262	150	66	25
357	260	129	62	33	10

Supplemental Figure 3: Estimated survival by cytopenia trajectory, adjusted by characteristics that statistically significantly differed across groups*

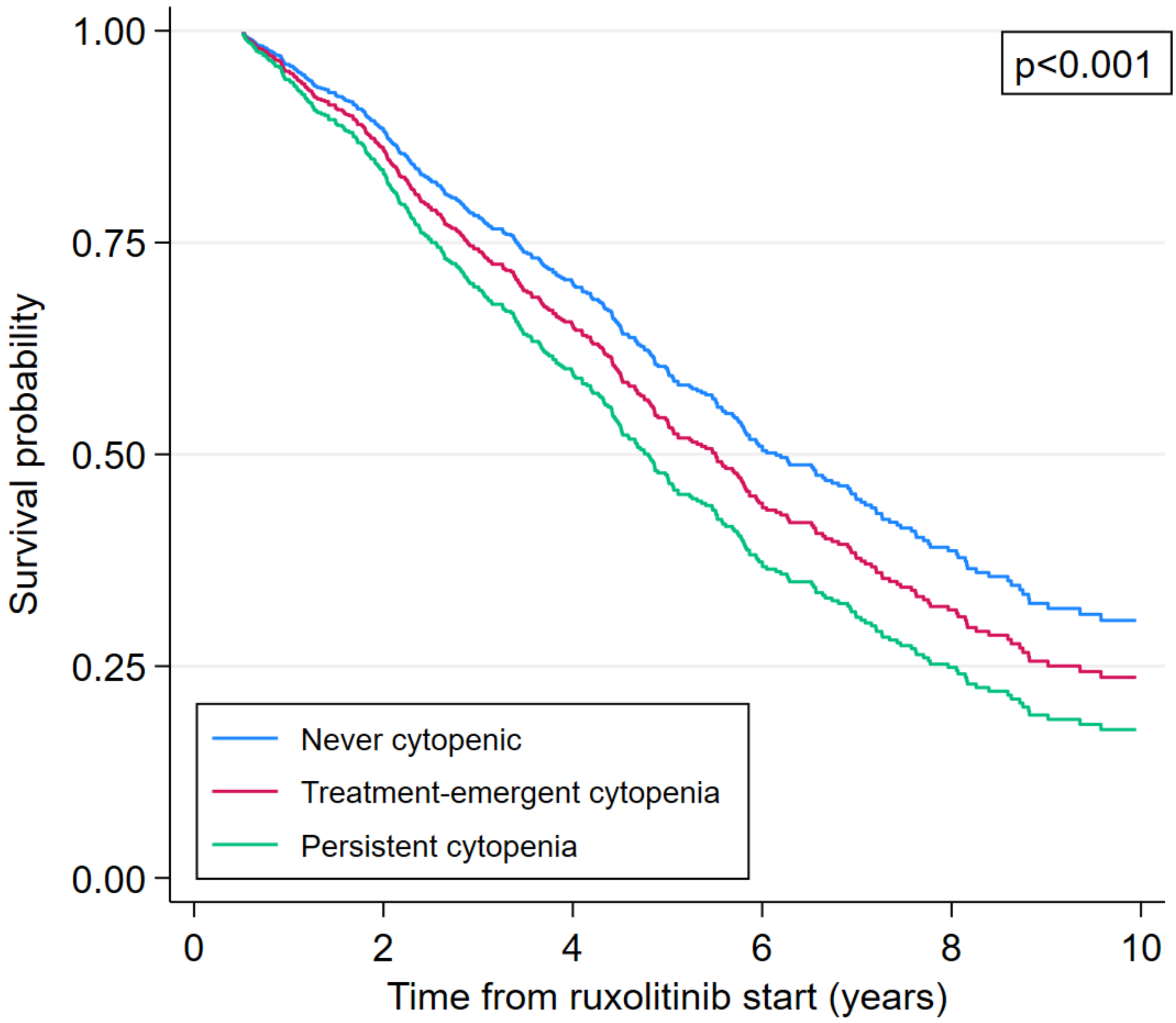

*Supplemental Figure 3: *adjusted by age ≥65, status of primary myelofibrosis, status of overt myelofibrosis, bone marrow fibrosis ≥3, ruxoliinib starting dose, hemoglobin level <10 g/dL, leukocytes count <4 x10^9/L, platelets count <100 x10^9/L, peripheral blasts ≥1%, spleen length >10 cm below costal margin, total symptoms score >20.*

Supplemental Figure 4: Estimated Survival by treatment-emergent cytopenia at 6 months adjusted by characteristics that statistically significantly differed across groups*

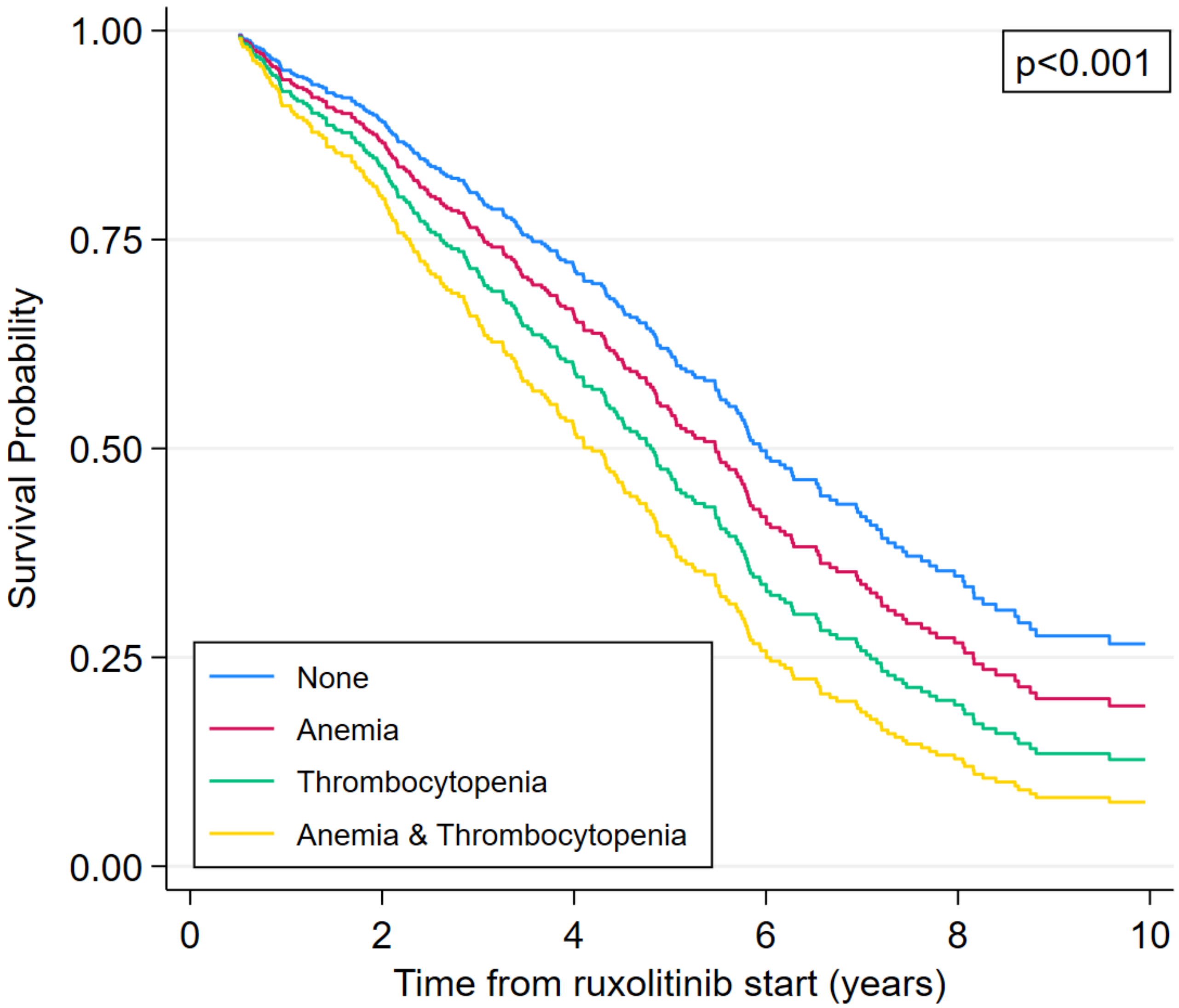

*Supplemental Figure 4: *adjusted by age ≥65, status of primary myelofibrosis, status of overt myelofibrosis, bone marrow fibrosis ≥3, ruxoliinib starting dose, hemoglobin level <10 g/dL, leukocytes count <4 x10^9/L, platelets count <100 x10^9/L, peripheral blasts ≥1%, spleen length >10 cm below costal margin, total symptoms score >20.*
